# GENDER DIFFERENCES IN THE RELATIONSHIP BETWEEN SEXUAL SATISFACTION AND SPIRITUAL WELL-BEING

**DOI:** 10.1093/geroni/igae098.2551

**Published:** 2024-12-31

**Authors:** Vivian Weiqun Lou, Clio Yuen Man Cheng

**Affiliations:** The University of Hong Kong, Hong Kong, China (People’s Republic); The University of Hong Kong, Hong Kong, China (People’s Republic)

## Abstract

Recognizing the significance of sexual activity in the holistic health of older adults, this research is the first to explore how sexual satisfaction contributes to the broader well-being dimensions, particularly spiritual well-being. Guided by an integration of the attachment theory and the spiritual well-being of Chinese older adults’ framework, we conducted a longitudinal study with 290 married young-olds aged 50 to 75 years. Participants completed self-reported measures assessing sexual satisfaction at baseline and three dimensions of spiritual well-being at 1-year follow-up. Linear regression analysis results indicate gender differences emerged in the associations between sexual satisfaction and these components of spiritual well-being. For the meaning of life, while males did not exhibit a significant association with sexual satisfaction, females demonstrated a positive association, suggesting that higher sexual satisfaction was linked to a greater sense of meaning in life (β =.728, SE =.365, t(1) = 1.997, p = 0.047). In terms of transcendence, no significant associations were found between sexual satisfaction and this component of spiritual well-being for either gender. Regarding overall spiritual well-being, males did not show a significant association with sexual satisfaction, while females displayed a positive association, indicating that higher sexual satisfaction was associated with higher levels of spiritual well-being (β = 1.175, SE = 0.410, t(1) = 2.868, p = 0.004). The findings underscore the significance of considering sexual satisfaction as a factor influencing spiritual well-being in older adults. Moreover, considering gender-specific patterns in future studies and interventions development for holistic well-being is warranted.

